# Acoustic, Thermal, and Mechanical Performance of Polymer-Coated Pumice Aggregate Lightweight Concretes

**DOI:** 10.3390/polym17243248

**Published:** 2025-12-06

**Authors:** Özlem Sallı Bideci, Alper Bideci, Ashraf Ashour, Amir Khan

**Affiliations:** 1 Department of Architecture, Faculty of Art, Design and Architecture, Düzce University, Düzce 81620, Türkiye; alperbideci@duzce.edu.tr; 2 Faculty of Engineering and Digital Technologies, University of Bradford, Bradford BD7 1DP, UK; a.s.ashour@bradford.ac.uk (A.A.); a.khan117@bradford.ac.uk (A.K.)

**Keywords:** polymer-coated pumice aggregate, acoustic performance, thermal properties

## Abstract

Pumice aggregate, with its highly porous structure, offers excellent lightweight and insulating characteristics; however, its excessive water absorption and weak interfacial bonding often limit its mechanical and durability performance in concrete applications. To overcome these drawbacks, this study developed a polymer-coated pumice aggregate (PCPA) concrete by applying a thin polyester layer onto the aggregate surface to enhance matrix–aggregate adhesion and reduce permeability. The mechanical, thermal, and acoustic performances of PCPA were systematically evaluated. Results revealed that polyester coating led to a notable improvement in compressive strength (up to 25%) and significantly reduced weight loss after freeze–thaw cycles. Furthermore, PCPA samples exhibited enhanced resistance to thermal degradation, maintaining structural stability even at 600 °C, and achieved a 40% higher sound absorption coefficient at 630 Hz compared to uncoated pumice concrete. These findings demonstrate that polyester coating effectively addresses the inherent limitations of pumice concrete, offering a promising approach for producing lightweight concretes with superior durability and multifunctional performance.

## 1. Introduction

Lightweight aggregates (LWAs), characterized by their lower bulk density compared to conventional aggregates, account for nearly 70% of the total concrete volume [1,2]. Both natural LWAs—such as pumice, diatomite, slag, sawdust, palm kernel shells, and bottom ash—and synthetic ones—such as expanded shale, perlite, slate, and slag—have been extensively studied for use in lightweight aggregate concrete (LWAC) [3,4,5,6,7,8,9,10]. The resulting concretes exhibit diverse physical and mechanical behaviors, which depend largely on the type and properties of the lightweight aggregate used [11].

LWACs are known for their superior frost resistance, fire resistance, and acoustic performance compared to conventional concretes [12,13,14,15,16]. However, their increased porosity and water absorption capacity often reduce their durability under severe environmental conditions. One of the primary deterioration mechanisms is frost damage, where water expansion upon freezing (by approximately 8–9%) generates tensile stresses that may exceed the tensile strength of concrete, leading to cracking and progressive material degradation [17]. Studies have shown that as the proportion of LWAs increases, the freeze–thaw resistance of concrete tends to decrease [14,18,19]. For example, mixtures containing 100% pre-saturated pumice aggregates exhibited the greatest strength reduction, while partial replacement levels (e.g., 50%) led to less deterioration [20].

Another key performance indicator of LWAC is its resistance to elevated temperatures [21,22]. Research has revealed that fiber-reinforced pumice concretes may retain or even improve strength at moderate temperatures (e.g., 250–350 °C), though strength significantly declines at higher levels (≥450 °C) [22]. Similarly, Amin et al. [23] and Bahrami and Nematzadeh [24] reported that lightweight concretes experienced considerable cracking and compressive strength loss (up to 56%) when exposed to 600 °C. Sancak et al. [25] found that both lightweight and normal-weight concretes deteriorated rapidly above 800 °C, with conventional concrete showing more pronounced degradation. These findings highlight the importance of enhancing the thermal stability of lightweight concretes for broader structural applications.

The acoustic behavior of LWAC also distinguishes it from conventional concrete. Sound absorption in concrete depends on its internal pore structure, where higher void content generally improves sound insulation [26,27,28]. However, some modifications—such as paraffin impregnation—may reduce this effect [28]. Studies replacing conventional aggregates with pumice stone have shown improved sound insulation [29], and the incorporation of rubber crumbs further enhances sound absorption by up to 228% depending on particle size [30]. Despite these advancements, challenges remain regarding the balance between lightweight, mechanical strength, and durability. One promising approach involves surface modification of LWAs to improve interfacial bonding and reduce water absorption. Among possible coating materials, polyester stands out due to its strong adhesion, chemical resistance, and thermal stability.

Accordingly, this study aims to develop and evaluate polymer-coated pumice aggregate (PCPA) concrete, where pumice aggregates are coated with polyester resin to enhance performance. The research investigates the effects of coating on physical properties, mechanical strength, freeze–thaw durability, thermal resistance, and sound absorption capacity. Through a comprehensive experimental program—including SEM-EDS analyses—this study seeks to address the limitations of uncoated pumice concretes and contribute a novel, performance-oriented approach to the design of durable and multifunctional lightweight concretes.

## 2. Materials and Methods

### 2.1. Materials

**Figure 1:**
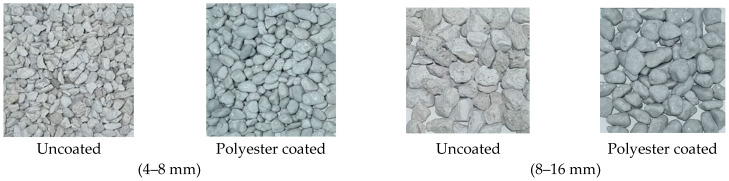
Polyester uncoated and coated pumice aggregates.

In this study, CEM I 42.5/R cement, conforming to TS EN 197-1 standards [31], was utilized as the binder. Fine aggregates comprised pumice with a particle size of up to 4 mm, supplied by Bimsblok from Nevşehir, Türkiye. Coarse aggregates included both uncoated and polyester-coated pumice aggregates in the particle size ranges of 4–8 mm and 8–16 mm, as depicted in Figure 1.

**Figure 2:**
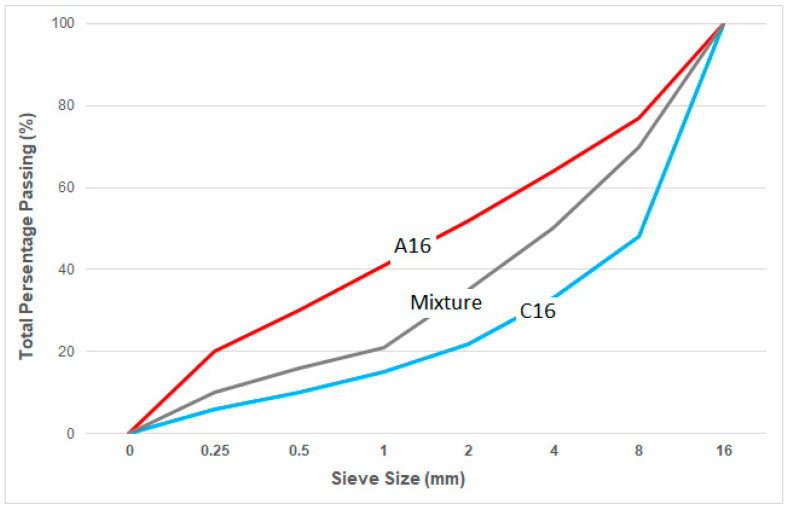
Granulometry curve of aggregates.

The density and water absorption properties of the aggregates were measured in accordance with the TS EN 1097-6 standard. For the fine aggregates, the specific gravity was determined to be 1540 kg/m^3^, the loose bulk density was 500 kg/m^3^, and the water absorption rate was 48.2%. In the case of coarse aggregates, the specific gravities of the uncoated pumice were 920 kg/m^3^ for the 4–8 mm size and 940 kg/m^3^ for the 8–16 mm size, with loose bulk densities of 440 kg/m^3^ and 390 kg/m^3^, respectively. The water absorption rates for the uncoated pumice aggregates were 35.2% for the 4–8 mm size and 27.4% for the 8–16 mm size. For the polyester-coated pumice aggregates, the specific gravities were 1020 kg/m^3^ (4–8 mm) and 1100 kg/m^3^ (8–16 mm), with corresponding loose bulk densities of 635 kg/m^3^ and 595 kg/m^3^. Water absorption rates for the coated aggregates were significantly lower, at 1.9% for 4–8 mm and 1.4% for 8–16 mm. Aggregate particle size distributions were assessed according to the TS 706 EN 12620 [32] standard, and the granulometric curve of the aggregate mixtures is given in Figure 2.

In order to prevent the pumice aggregates from clumping together during the coating process, marble powder was utilized. The polyester employed in this study is an orthophthalic-based, general-purpose unsaturated casting resin, characterized by low reactivity and medium viscosity. The chemical compositions of the materials used in the study are presented in Table 1.

### 2.2. Mix Proportions

In this research, a total of nine series of lightweight concrete were developed by substituting uncoated pumice aggregates in sizes 4–8 mm and 8–16 mm with polyester-coated pumice aggregates at replacement levels of 0%, 50%, and 100%. The cement dosages varied at 200, 250, and 300 kg/m^3^. The series using only uncoated pumice aggregates are designated as REF, those with 50% uncoated and 50% polyester-coated pumice aggregates are labeled as PC50, and the series with 100% polyester-coated pumice aggregates are marked as PC100, with the specific dosages noted alongside the codes. Three specimens were tested for each experimental condition. The detailed mixture design for each experimental series is summarized in Table 2.

### 2.3. Testing Procedure

This study consists of four experimental stages. In the first stage, a total of 162 cubic samples measuring 100 × 100 × 100 mm and 27 cylindrical samples with a diameter of 100 mm and a height of 50 mm were produced based on the mixture calculations for the 9 series and were left for a 28-day water curing period. In the second stage, the unit weight and water absorption of the PCPA concretes were determined in accordance with TS EN 12390-7 standards, and the compressive strengths were measured according to TS EN 12390-3 standards. For the freeze–thaw (100 cycles) test, the samples were subjected to freeze–thaw cycles in a chamber with a temperature range of +4 °C to –18 °C, completing each cycle in accordance with ASTM C 310 standard. For the high temperature test, all samples except those at 20 °C (Control) were placed in an oven where the temperature was increased in 5 °C increments to 200 °C, 400 °C, and 600 °C. Once the desired temperature was reached, the samples were held at a constant temperature for 1 hour before being allowed to cool after the oven was turned off. Dry unit weight and compressive strength tests were performed on the samples before and after exposure to high temperatures.

**Figure 3:**
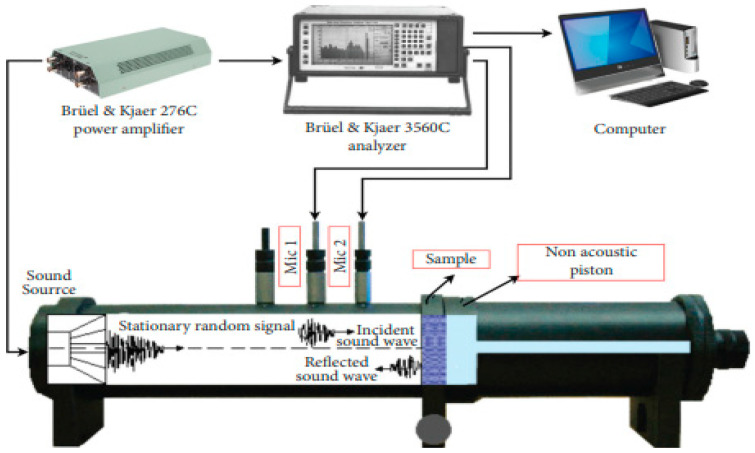
Impedance tube setup for testing sound absorption properties [35].

In the third stage, the internal structure of the samples was examined using Scanning Electron Microscopy (SEM) images obtained with an FEI Quanta Feg 250 model variable pressure instrument (FEI, Hillsboro, OG, USA) and Energy Dispersive Spectrometry (EDS) analyses were conducted using an EDAX Apollo X model device (EDAX, Mahwah, NJ, USA). In the fourth and final stage, the sound absorption performance of the samples was determined using the impedance tube method in accordance with ISO 10534-2 [33] standards. The measurement was carried out using a Standard Bruel and Kjaer type 4206 impedance tube (Bruel and Kjaer, Nærum, Danmark) with a diameter of 100 mm. The impedance tube speaker was driven by a Bruel and Kjaer power amplifier of type 2706, as shown in Figure 3. For acoustic absorption measurements a closed end tube was used with a hard backing. For the closed tube condition, a broadband signal was applied to measure the absorption coefficient of the concrete samples. A single microphone method was employed to measure the broadband signal at each microphone port, which eliminated the need for calibration of the microphone at each location on the tube. Two microphone ports were utilized during this experiment, and the same microphone probe was used to measure the signal sequentially at each location. Signals recorded at these channels were analyzed to determine the absorption coefficient of the tested sample [34].

## 3. Results

### 3.1. Dry Unit Weight, Water Absorption and Compressive Strength

**Figure 4:**
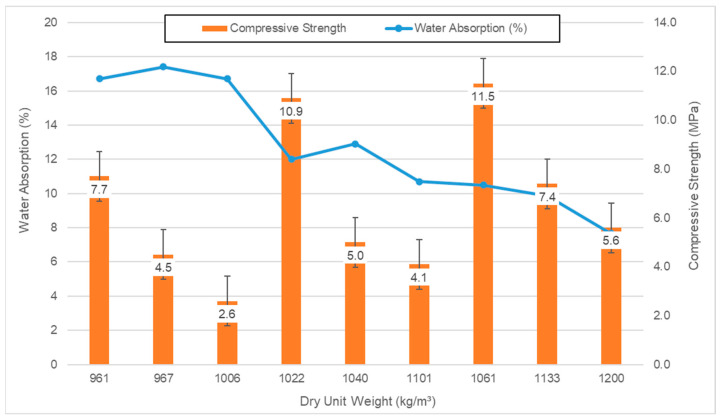
Dry unit weights, water absorption rates and compressive strength of the samples.

The results of water absorption rates, and compressive strength against the dry unit weight of hardened concrete samples are shown in Figure 4. The application of polyester coating to aggregates and the increase in cement dosage led to an increase in the unit weight and compressive strength of the mixtures, while conversely causing a decrease in the water absorption rates.

Upon examining Figure 4, the lowest dry unit weight value of 961 kg/m^3^ was exhibited by REF-D200 samples, while the highest dry unit weight value of 1200 kg/m^3^ was obtained from PC100-D300 samples. In the study, the unit weight values of all series increased with the application of polyester coating to the pumice aggregates and the increase in cement dosage. All series remained below the upper limit of dry unit weight (2000 kg/m^3^) specified for lightweight concretes in the literature [36]. According to TS EN 206-1, REF-D200 and PC50-D200 are classified in the D1.0 density class (≥800 and ≤1000), while all other series fall into the D1.2 density class (≥1000 and ≤1200). These results are consistent with studies conducted by other researchers [37,38,39,40,41,42].

In the water absorption test results, the lowest water absorption value of 7.7% was recorded for PC100-D300 samples, and the highest water absorption value of 17.4% was found in REF-D200 samples. It was determined that polyester coating on pumice aggregates and the increase in dosage resulted in a decrease in water absorption values [43,44].

The lowest compressive strength value of 2.6 MPa was obtained from PC100-D200 samples, while the highest compressive strength value of 11.5 MPa was achieved by REF-D300 samples. It was observed that compressive strength values increased with cement dosage and decreased with the amount of PCPA used [44]. According to ASTM C09 [45] standard, the compressive strength of insulating concrete is less than 10 MPa, while the compressive strength of semi-structural lightweight concrete is around 15 MPa. Based on this, it was determined that the REF-D250 and REF-D300 dosage series fall into the semi-structural lightweight concrete class, while all other series fall into the insulating concrete class. 

The decrease in compressive strength at higher PCPA contents is mainly attributed to the weaker interfacial transition zone (ITZ) between the polymer-coated aggregates and the cement matrix. Although the polymer coating improves durability by reducing water absorption, it also forms a smoother surface, which limits mechanical interlocking and bond strength. SEM observations further confirm this, showing a less compact hydration structure and slight separation at the ITZ of coated samples.

### 3.2. Freeze–Thaw Resistance Testing

**Figure 5:**
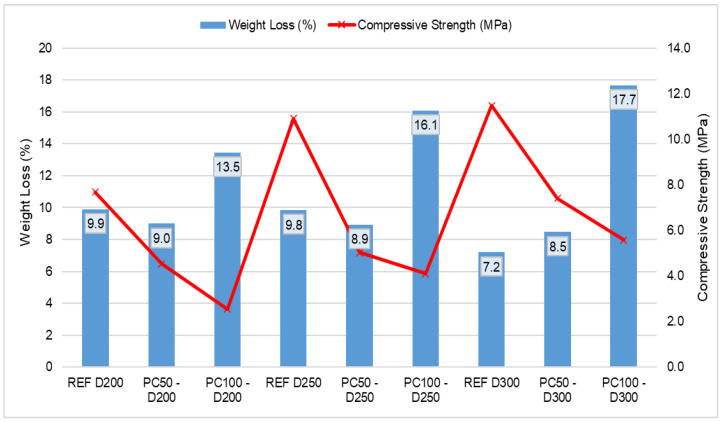
Weight loss and compressive strength test results of concrete samples after freeze–thaw resistance testing.

The weight loss and compressive strength test results after 100 freeze–thaw cycles of the produced test samples are shown in Figure 5. This Figure 5 indicates that the lowest weight loss of 7.2% was obtained from the REF D300 series, while the highest weight loss of 17.7% was demonstrated by the PC100 D300 series. When evaluating the weight loss values after freeze–thaw resistance test for different PCPA (0%, 50%, 100%) usage compared to the REF samples, it was found that in the 200 and 250 cement dosage series, PC50 samples showed a 10.0% decrease, while PC100 samples showed increases of 26.7% and 39.1%, respectively. In the 300 cement dosage series, PC50 and PC100 samples experienced weight losses of 15.3% and 59.3%, respectively. When assessed according to cement dosages, the 200 cement dosage series had weight loss increases of 1.0% and 27.3% for REF samples, 1.1% and 5.6% for PC50 samples, and 19.26% and 31.1% for PC100 samples after freeze–thaw resistance compared to the 250 and 300 cement dosage series. It was found that in all series with 50% PCPA, the weight loss due to freeze–thaw resistance decreased with increasing cement dosage, whereas in all series with 100% PCPA, the weight loss increased despite the higher cement dosage. This situation can be attributed to the gradual widening of internal micro-cracks and pores in the concrete and the slow degradation of the sample surface with increasing freeze–thaw cycles [46].

Regarding the compressive strengths of the samples after 100 cycles, it was determined that the lowest compressive strength of 2.7 MPa was obtained from PC100 D200 samples, while the highest compressive strength of 11.7 MPa was recorded for REF D300 samples. Evaluating the compressive strength values after freeze–thaw resistance for different PCPA (0%, 50%, 100%) usage, it was observed that REF D200 samples decreased by 41.0% and 67.5%, REF D250 samples decreased by 45.1% and 59.3%, and REF D300 samples decreased by 21.4% and 38.5%, respectively. When assessed according to cement dosages, the 200 cement dosage series showed increases of 36.1% and 41.0% in REF samples, 26.5% and 87.8% in PC50 samples, and 70.4% and 166.7% in PC100 samples compared to the 250 and 300 cement dosage series. The study found that in all series, the compressive strength after freeze–thaw resistance increased with cement dosage, while the compressive strengths decreased with increasing PCPA usage [47]. The increase in compressive strength after freeze–thaw cycles may be related to the infiltration of excess water into the pores of lightweight aggregates during freezing events as explained in the literature [48,49].

### 3.3. Temperature Impact on Weight Loss and Strength

**Figure 6:**
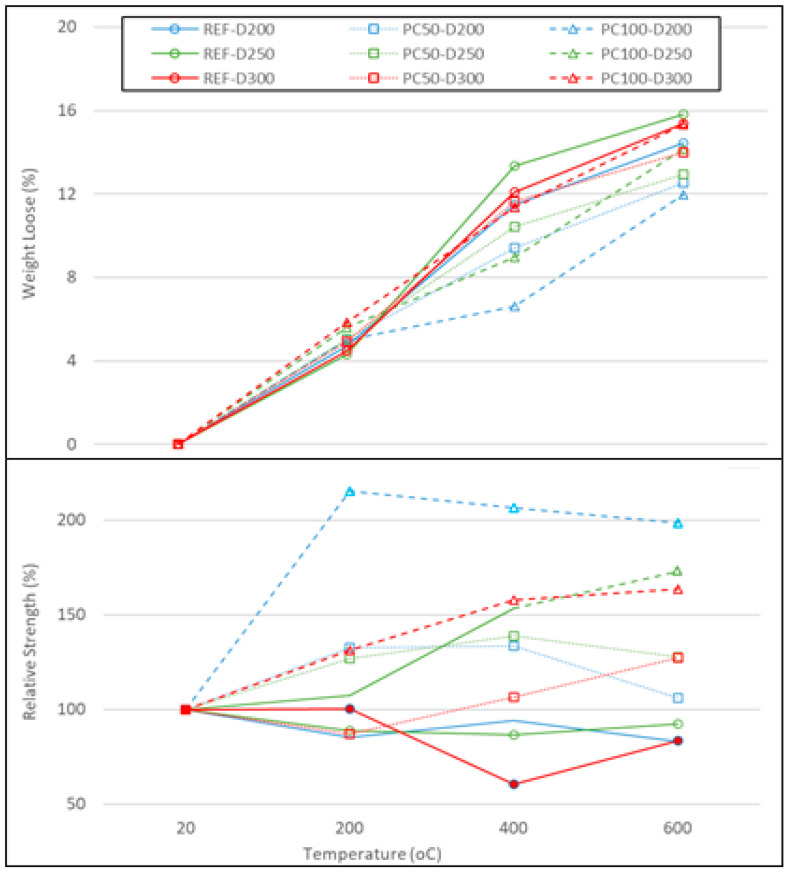
Weight losses and relative strength of concrete specimens subjected to elevated temperature.

The weight losses of samples under high temperatures and their relative strength are shown in Figure 6. The upper plot illustrates the percentage of weight loss as a function of temperature, while the lower plot represents the relative strength of the samples at corresponding temperature levels. The data reveal distinct trends in material properties, which are influenced by both thermal exposure and compositional differences.

In the upper plot, the weight loss percentage of the samples generally shows an increasing trend with rising temperature, reflecting the thermal decomposition or evaporation of volatile compounds. Notably, the samples labeled REF-D300 and PC50-D300 exhibit the highest weight loss, especially beyond 400 °C, suggesting a greater susceptibility to thermal degradation compared to other samples. Conversely, samples with labels PC100-D200 and PC100-D250 show relatively lower weight loss, indicating better thermal stability up to 600 °C.

The lower plot presents the relative strength variations of the samples as a function of temperature. At 20 °C, all samples begin at a relative-strength value of 100%, serving as a baseline. However, as the temperature rises, there is a noticeable divergence in behavior. The samples labeled PC100-D200 demonstrate a significant increase in relative strength up to 400 °C, which may result from temporary densification caused by moisture evaporation or partial softening and sintering of the polyester coating that enhances the interfacial bond between the coated aggregates and the cement paste. In contrast, samples such as REF-D300 show a continuous decline in relative strength, particularly after 400 °C, implying a reduction in structural integrity due to the breakdown of hydration products and loss of cohesive forces.

These results indicate that the thermal stability and strength retention of the samples are strongly dependent on their composition and microstructural properties, with certain formulations exhibiting better performance at elevated temperatures. The distinct trends observed in weight loss and relative strength emphasize the complex interaction between temperature, material composition, and mechanical behavior, which is critical for understanding their potential in thermally demanding applications.

### 3.4. Scanning Electron Microscopy (SEM) and Energy Dispersive Spectroscopy (EDS) Analyses

**Figure 7:**
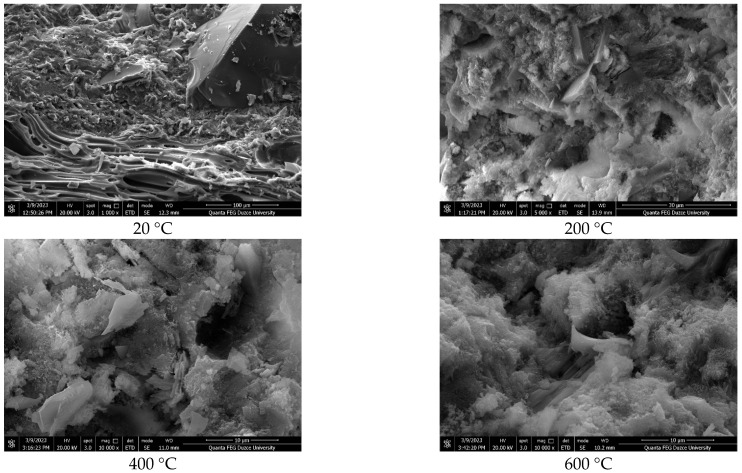
SEM images of REF D250.

**Figure 8:**
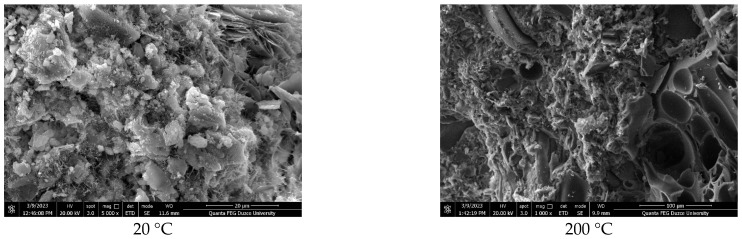

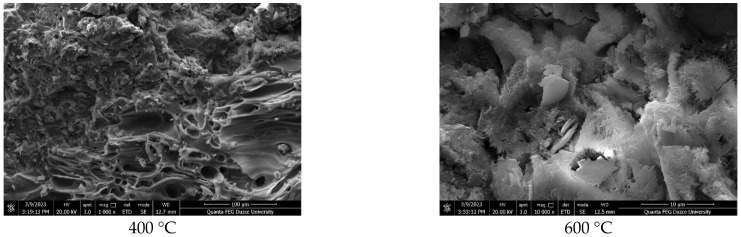
SEM images of PC50 D250.

**Figure 9:**
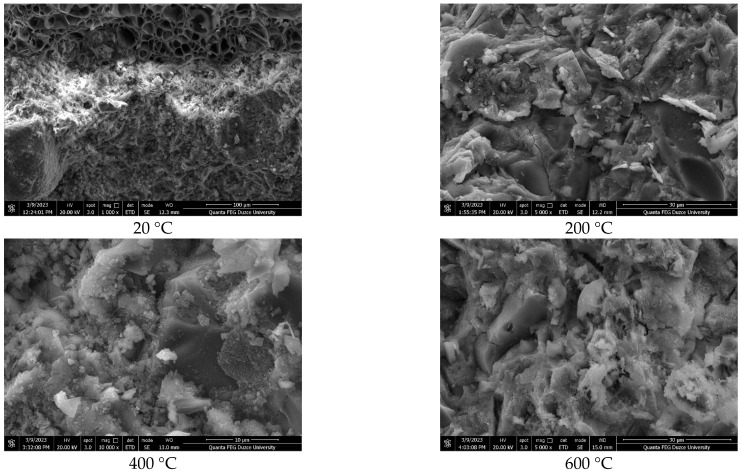
SEM images of PC100 D250.

SEM images of the lightweight concrete samples produced with polyester-coated and uncoated pumice aggregates are shown in Figure 7, Figure 8 and Figure 9, and EDS analyses are provided in Table 3.

The SEM analyses indicate that the samples comprise lightweight aggregates with a glassy morphology [42]. The pumice aggregates display tube-like channels at the micropore scale and possess high porosity [50,51,52]. It is well recognized that the properties of the interface significantly affect the porosity and permeability of concrete, and that micro-cracking within the matrix phase plays a crucial role in these characteristics [53]. Additionally, the pore structure of concrete substantially influences its strength, with physical attributes such as porosity and density directly impacting mechanical properties. The marble powder used in the coating may modify the concrete’s pore structure, as illustrated in [53]. Moreover, it is important to note that the additives involved in the degradation of the polyester material, including their concentration and average particle size, have a considerable impact on the strength characteristics of the coating [54].

Table 3 presents the elemental analysis obtained via EDS, highlighting the elemental peaks associated with the mineral additive, marble powder. Additionally, hydrated phases such as calcium silicate hydrate (C–S–H), portlandite, and ettringite, along with unhydrated cement particles, were identified.

### 3.5. Acoustic Properties

**Figure 10:**
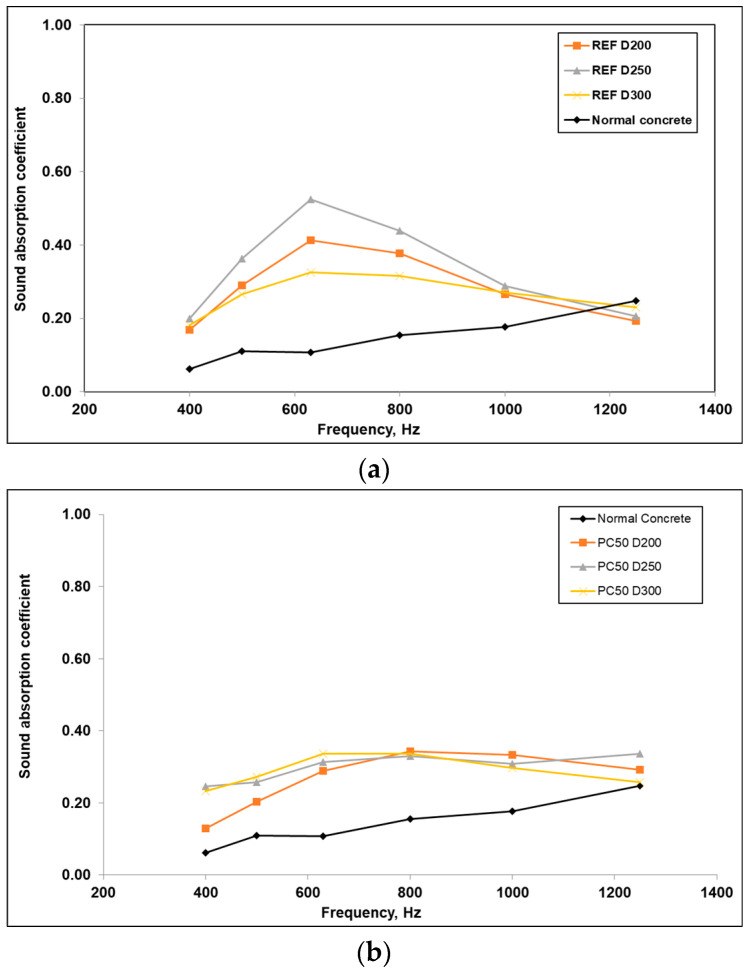

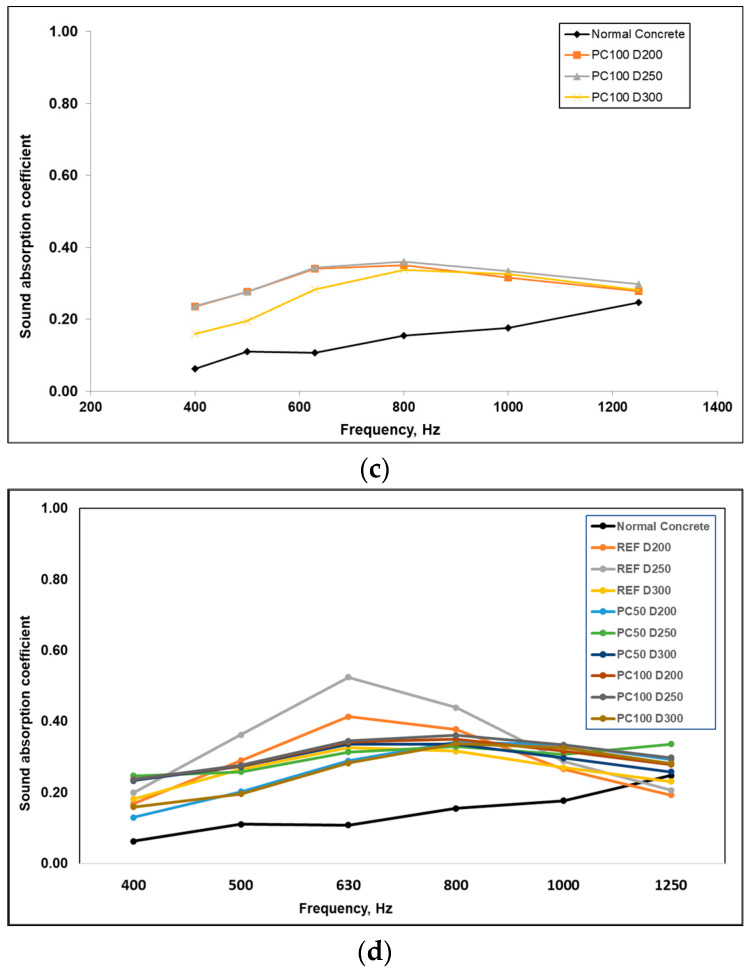
The acoustic absorption coefficient results. (**a**) Acoustic absorption coefficient of PCPA (REF). (**b**) Acoustic absorption coefficient of PCPA (PC50). (**c**) Acoustic absorption coefficient of PCPA (PC100). (**d**) Acoustic absorption coefficient of PCPA all series.

The acoustic absorption coefficient of a material measures its ability to absorb sound. A material with excellent absorption has a value close to 1.0, whereas a material that reflects sound well has a value close to 0. Normal concrete mixtures typically have values ranging from 0.05 to 0.10 [55]. In this study, results from frequencies below 400 Hz were not evaluated. The acoustic absorption coefficient results at various frequencies are presented in Figure 10: Figure 10a for REF samples, Figure 10b for PC50 samples, Figure 10c for PC100 samples, and Figure 10d for all samples.

Figure 10 indicates that the sound absorption coefficients for all PCPA series initially increases with frequency and then decreases. This trend is particularly evident with the maximum absorption coefficient reached at a frequency of 630 Hz. The REF D300 series is found to have the highest sound absorption coefficient, approximately 0.7 at 630 Hz. Among the other PCPA series, the sound absorption performance of REF and PC50 series are quite similar, while the PC100 series generally exhibits slightly lower absorption. The sound absorption coefficient of normal concrete is significantly lower compared to other materials across all frequencies. Specifically, at lower frequencies (400 Hz), the sound absorption coefficient of normal concrete is about 0.1, whereas the coefficients for the PCPA series are above 0.2. This indicates that the PCPA series outperform normal concrete in terms of acoustic performance.

## 4. Conclusions

This study investigated the mechanical, physical, thermal, and acoustic performance of polymer-coated pumice aggregate (PCPA) concrete, focusing on the effects of polyester coating and varying cement dosages. The findings collectively demonstrate that polyester coating significantly enhances certain material properties of lightweight concrete, although the extent of improvement depends on both the coating ratio and cement content.

Polyester coating improved the dry unit weight and compressive strength of the concrete, confirming its effectiveness in enhancing interfacial bonding and reducing aggregate porosity. In particular, higher cement dosages further contributed to reduced water absorption, with the lowest absorption observed in the PC100-D300 mixture. This improvement indicates that polymer coating enhances durability and moisture resistance, which are critical for long-term performance.

However, increasing the proportion of polymer-coated aggregates resulted in a noticeable decrease in compressive strength, especially at lower cement dosages, highlighting the importance of optimizing the balance between lightweight properties and mechanical performance. According to classification standards, the REF-D250 and REF-D300 series qualify as semi-structural lightweight concrete, while the remaining mixes fall under insulating concrete—consistent with their densities and strengths.

Durability assessments under freeze–thaw cycles revealed that while the reference concretes retained strength effectively, the 100% coated series experienced greater weight and strength losses. These results suggest that although the coating improves certain properties, excessive coating content may hinder freeze–thaw resistance by altering the aggregate–matrix interface.

At elevated temperatures, all samples exhibited increasing weight loss with temperature rise. The 50% coated mixtures performed slightly better than the uncoated ones, indicating that partial coating can provide improved thermal stability without compromising integrity. Microstructural analyses confirmed that polyester coating modifies the pore structure and improves bonding at the interface, while EDS results verified the presence of mineral additives contributing to strength development.

From an acoustic standpoint, the PCPA series showed markedly higher sound absorption, particularly around 630 Hz, compared to normal concrete. This confirms the potential of polymer-coated pumice concretes for applications requiring lightweight, thermally resistant, and acoustically efficient materials.

In summary, polyester coating of pumice aggregates offers a practical approach to improving the multifunctional performance of lightweight concretes. The optimal balance between coating ratio and cement dosage is crucial for achieving both structural adequacy and enhanced durability. Future studies should further investigate coating thickness optimization and the long-term environmental performance of polymer-coated lightweight concretes.

## Figures and Tables

**Table 1:** Chemical analysis of the materials used (%).

Chemical Composition	SiO_2_	Al_2_O_3_	Fe_2_O_3_	CaO	MgO	K_2_O	Na_2_O	SO_3_	Loss Ignition
**Pumice**	68.70	14.90	2.80	2.85	0.90	2.65	3.90	-	4.25
**CEM I 42.5/R**	19.60	5.10	2.50	63.85	1.16	0.70	0.28	3.15	3.92
**Marble** **Powder**	-	-	0.02	31.18	23.33	0.01	0.51	-	44.76

**Table 2:** Concrete mixture proportions (1 m^3^).

Cement Dosage (kg/m^3^)	PCPA Replacement Ratio (%)	Sample Notation	Pumice Aggregate (kg)						Cement (kg)	Water * (kg)
			Sieve Aperture							
			0–1 (mm)	1–4 (mm)	4–8 (mm)		8–16 (mm)			
					Coated	Non-Coated	Coated	Non-Coated		
**200**	**0%**	**REF D200**	167.10	668.38	0.00	230.60	0.00	153.73	200	100
	**50%**	**PC50 D200**	167.10	668.38	152.89	115.30	91.91	76.86	200	100
	**100%**	**PC100 D200**	167.10	668.38	305.79	0.00	183.81	0.00	200	100
**250**	**0%**	**REF D250**	158.88	635.48	0.00	219.25	0.00	146.16	250	125
	**50%**	**PC50 D250**	158.88	635.48	145.37	109.62	87.38	73.08	250	125
	**100%**	**PC100 D250**	158.88	635.48	290.73	0.00	174.76	0.00	250	125
**300**	**0%**	**REF D300**	150.64	602.58	0.00	207.89	0.00	138.60	300	150
	**50%**	**PC50 D300**	150.64	602.58	137.84	103.94	82.85	69.29	300	150
	**100%**	**PC100 D300**	150.64	602.58	275.68	0.00	165.72	0.00	300	150

* In addition, before the concrete mix, the pumice aggregates were prewetted.

**Table 3:** EDS results of REF D250, PC50 D250 and PC100 D250 samples.

REF D250						
Temperature	Si	Al	Ca	Na	C	O
**20 °C**	11.24	7.50	35.20	0.72	2.56	38.99
**200 °C**	19.75	2.82	43.43	-	3.64	25.50
**400 °C**	17.84	2.86	49.58	0.52	-	25.33
**600 °C**	7.12	6.02	62.87	-	-	19.71
**PC50 D250**						
**20 °C**	18.39	2.39	50.00	0.20	5.61	16.85
**200 °C**	19.89	3.07	48.55	0.44	-	24.61
**400 °C**	49.84	10.15	2.33	3.49	-	29.38
**600 °C**	5.35	6.73	53.86	1.11	4.91	18.02
**PC100 D250**						
**20 °C**	38.41	7.97	2.63	2.03	-	45.06
**200 °C**	12.28	1.71	56.65	-	3.22	22.26
**400 °C**	19.94	3.90	40.03	0.91	5.80	24.15
**600 °C**	17.03	2.93	33.97	0.47	4.92	36.65

## Data Availability

Data are contained within the article.
